# The Small GTPase FgRab1 Plays Indispensable Roles in the Vegetative Growth, Vesicle Fusion, Autophagy and Pathogenicity of *Fusarium graminearum*

**DOI:** 10.3390/ijms23020895

**Published:** 2022-01-14

**Authors:** Yanping Yuan, Meiru Zhang, Jingjing Li, Chengdong Yang, Yakubu Saddeeq Abubakar, Xin Chen, Wenhui Zheng, Zonghua Wang, Huawei Zheng, Jie Zhou

**Affiliations:** 1 Fujian Universities Key Laboratory for Plant-Microbe Interaction, College of Life Sciences, Fujian Agriculture and Forestry University, Fuzhou 350002, China; yyuan97@163.com (Y.Y.); zmr17629780871@163.com (M.Z.); 13414927525@163.com (J.L.); Chengdy@hainanu.edu.cn (C.Y.); ay.saddeeq@yahoo.com (Y.S.A.); wangzh@mju.edu.cn (Z.W.); 2 Department of Biochemistry, Faculty of Life Sciences, Ahmadu Bello University, Zaria 810211, Nigeria; 3 State Key Laboratory of Ecological Pest Control for Fujian and Taiwan Crops, College of Plant Protection, Fujian Agriculture and Forestry University, Fuzhou 350002, China; danhinm1996@outlook.com (X.C.); wenhuiz@fafu.edu.cn (W.Z.); 4 Marine and Agricultural Biotechnology Laboratory, Institute of Oceanography, College of Geography and Oceanography, Minjiang University, Fuzhou 350108, China

**Keywords:** *Fusarium graminearum*, FgRab1, constitutively active, dominant negative, vesicle transport, autophagy, pathogenicity

## Abstract

Rab GTPases are key regulators of membrane and intracellular vesicle transports. However, the biological functions of FgRab1 are still unclear in the devastating wheat pathogen *Fusarium graminearum*. In this study, we generated constitutively active (CA) and dominant-negative (DN) forms of *FgRAB1* from the wild-type PH-1 background for functional analyses. Phenotypic analyses of these mutants showed that FgRab1 is important for vegetative growth, cell wall integrity and hyphal branching. Compared to the PH-1 strain, the number of spores produced by the *Fgrab1DN* strain was significantly reduced, with obviously abnormal conidial morphology. The number of septa in the conidia of the *Fgrab1DN* mutant was fewer than that observed in the PH-1 conidia. *Fgrab1DN* was dramatically reduced in its ability to cause Fusarium head blight symptoms on wheat heads. GFP-FgRab1 was observed to partly localize to the Golgi apparatus, endoplasmic reticulum and Spitzenkörper. Furthermore, we found that FgRab1 inactivation blocks not only the transport of the v-SNARE protein FgSnc1 from the Golgi to the plasma membrane but also the fusion of endocytic vesicles with their target membranes and general autophagy. In summary, our results indicate that FgRab1 plays vital roles in vegetative growth, conidiogenesis, pathogenicity, autophagy, vesicle fusion and trafficking in *F. graminearum*.

## 1. Introduction

Ras related in brain (Rab) GTPases make up the largest group of the small GTPase family (called Ypt proteins in yeast) and function as molecular switches in the regulation of intracellular membrane trafficking in all eukaryotic cells [1,2,3,4]. Rab GTPases alternate between active GTP-bound and inactive GDP-bound states, which are facilitated by guanine nucleotide exchange factors (GEFs) and GTPase-activating proteins (GAPs), respectively [4]. Rab GTPases have a conserved GTP/GDP core and hydrolysis domain with highly conserved motifs, such as G1 (Walker A/P-loop; GxxxxG K[S/T]), which is implicated in the binding of the β- and γ-phosphate groups of nucleotides [5,6]. In fungi, the number of Ypt/Rab protein families is stable from 7 to 12 [4,7,8], of which Rab5/Ypt5, Rab7/Ypt7 and Rab8/Sec4 are well studied.

Rab GTPases have been systematically investigated in the filamentous fungus *Aspergillus nidulans*. The members of this protein family have been shown to be involved in multi-fungal intracellular trafficking processes, including exocytosis and endocytosis, and the Spitzenkörper of *A. nidulans* has been shown to harbor four Rab proteins, namely, Rab11, Sec4/Rab8, Rab6 and Rab1 [1]. In addition to those in *A. nidulans*, the Rab GTPases in other fungi, notably, *Ustilago maydis*, *Magnaporthe oryzae*, *Neurospora crassa* and *F. graminearum*, have also been well investigated [1,9].

Rab GTPases (including Rab8/Sec4, Rab7/Ypt7 and Rab5) have been well studied in the last ten years, especially in plant pathogenic fungi. In the hemibiotrophic pathogen *Colletotrichum orbiculare*, Sec4 was shown to localize to the necks of biotrophic primary hyphae, adjacent to the interface, and the deletion of *SEC4* reduced the virulence and disrupted effector delivery to the ring interface [10]. In *U. maydis*, the deletion of Rab5a reduced effector transcription and secretion, preventing the fungus from effectively colonizing plant tissues [11]. *BcSAS1*, the Rab8/SEC4-like gene, plays an important role in the development, protein secretion and virulence of *Botrytis cinerea* [12]. In *Fusarium verticillioides*, FvSec4 is necessary for fumonisin B1 biosynthesis and virulence [13]. In the rice blast fungus *M. oryzae*, Rab5A and Rab5B are essential for the development and pathogenicity of the fungus, but they have distinct biochemical and functional properties [14,15]; MoRab7/Ypt7 is required for membrane fusion during autophagy and pathogenicity [16], and it recruits the retromer cargo-sorting complex to the endosome [17]. Rab8/Sec4 is involved in vegetative growth and pathogenicity by regulating extracellular protein secretion [18]. In the plant fungal pathogen *Colletotrichum lindemuthianum*, CLPT1 (Sec4) is required for protein secretion and pathogenesis [19].

The above studies mainly focus on Rab5, Rab7/Ypt7 and Rab8/Sec4. However, the roles of Rab1 (Ypt1) in plant pathogenic fungi are still unclear. In yeast, *YPT1* is an essential gene [20,21], in which Asn121--IIe substitution within the gene product had a dominant lethal phenotype [22]. Ypt1 is involved in microtubule organization, where it functions in the secretion pathway, and it is required for transport to and through the Golgi apparatus [23]. In *Schizosaccharomyces pombe*, Ypt1 is essential for cell growth [24]. The overexpression of a dominant-negative allele of *YPT1* inhibits growth and aspartyl protease secretion in the pathogenic yeast *Candida albicans* [25]. In mammalian cells, Rab1 is required for the fusion of carrier vesicles, thereby mediating endoplasmic reticulum-to-Golgi transport [26]. However, the roles of Rab1/Ypt1 in plant pathogenic fungi are still unknown.

*F. graminearum* is a filamentous ascomycete and the causal agent of Fusarium head blight (FHB) in wheat worldwide [27,28,29,30]. *F. graminearum* infects wheat spikelets and colonizes the entire wheat head by growing through the rachis node at the bottom of each spikelet [31]. In addition, during the infection of *F. graminearum* on wheat florets, it forms specialized infection structures, such as lobate appressoria and infection cushions [32]. In addition to wheat, *F. graminearum* also infects maize, barley and other small grains [33,34,35,36]. *F. graminearum* infections not only result in severe yield losses but also contaminate the grains with various mycotoxins, imposing serious potential impacts on human and animal health [37]. In *F. graminearum*, Rab GTPases are essential for membrane trafficking-dependent growth, DON production and pathogenicity [9]; FgRab51 and FgRab52 regulate early endocytosis; FgRab7 regulates late endocytosis and vacuolar fusion; and FgRab8 and FgRab11 are required for polarized growth and secretion. Furthermore, FgVps9, FgSec2A and FgMon1 were identified as the guanine nucleotide exchange factors (GEFs) of FgRab5, FgRab8 and FgRab7, respectively [38,39,40]. FgGyp1 and FgMsb3 were demonstrated to act as the GTPase-activating proteins (GAPs) for FgRab1 and FgRab8, respectively [41,42], and they are both required for the SNARE protein FgSnc1-mediated fusion of vesicles with the plasma membrane.

Considering the fact that Rab1/Ypt1 is an essential gene, particularly in yeast, this study focused on investigating the functions of FgRab1 in the development and pathogenesis of *F. graminearum*. We found that the overexpression of a dominant-negative form of FgRab1 inhibits vegetative growth, conidiation, cell wall integrity, pathogenicity, autophagy and the transport of v-SNARE FgSnc1 from the Golgi to the plasma membrane in *F. graminearum*. Furthermore, FgRab1 localizes to the Golgi apparatus, endoplasmic reticulum and Spitzenkörper.

## 2. Results

### 2.1. Identification of FgRab1 in F. graminearum

**Figure 1:**
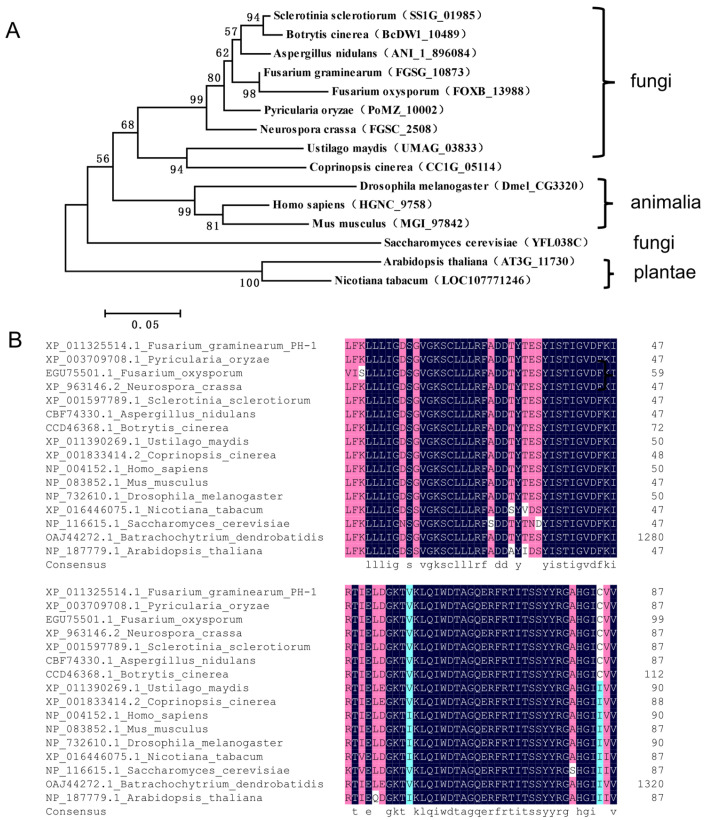
Bioinformatics analysis of Rab1 orthologs. (**A**) Phylogenetic analysis of Rab1 homologs in different species. (**B**) Multiple sequence alignment of Rab1 homologs.

We have previously identified *FgRAB1* (FGSG_10873) in the *F. graminearum* genome using the NCBI database (https://www.ncbi.nlm.nih.gov/, accessed on 13 January 2022) [9]. In the present study, we obtained the amino acid sequence of FgRab1 and its homolog proteins in 15 different species, and we used MEGA5 software to construct the phylogenetic tree of the Rab1 protein. We found that the Rab1 proteins are relatively conserved in most species, and FgRab1 is closest to *Fusarium oxysporum* in terms of evolutionary relationship (Figure 1A).

Previous studies have shown that many Rab proteins are characterized by the presence of five specifically conserved domains, namely, IGVDF (RabF1), KLQIW (RabF2), RFRSIT (RabF3), YYRGA (RabF4) and LVYDIT (RabF5) [43]. The C-termini of these proteins may have the membrane positioning signal motif XXXCC, XXCCX, XCCXX, CCXXX or XXCXC (where X is any amino acid), which can be prenylated to guide the Rab protein to anchor the target membrane [43,44,45]. The amino acid sequences of Rab1 proteins from 15 species were compared using DNAMAN software, and the sequence alignment results suggest that most of the Rab1 proteins have the above conserved domains (Figure 1B). Furthermore, previous studies revealed that Rab proteins generally possess GTP- and GDP-binding sites. Herein, our amino acid sequence comparison of the Rab1 proteins from 15 species showed that the GTP- and GDP-binding sites were very conservative in different species (Figure 1B).

### 2.2. FgRab1 Is Critical for Vegetative Growth and Is Involved in the Maintenance of Cell Wall Integrity of F. graminearum

**Figure 2:**
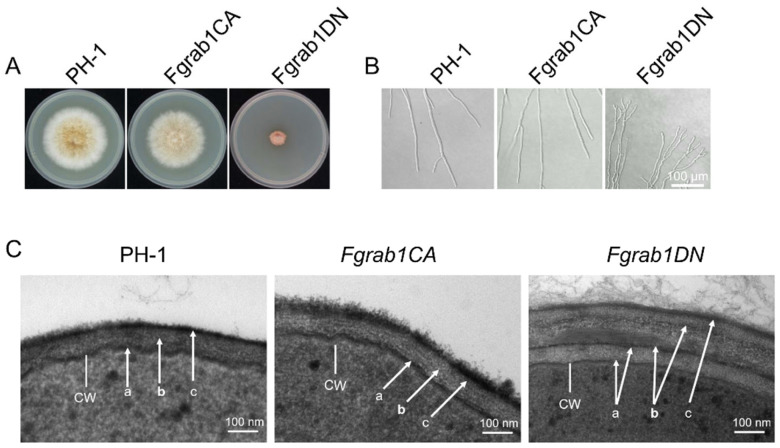
FgRab1 is important for vegetative growth and growth polarity in *F. graminearum*. (**A**) Colony morphology of the wild-type PH-1, *Fgrab1CA* and *Fgrab1DN* strains. (**B**) Mycelial morphology of PH-1, *Fgrab1CA* and *Fgrab1DN*. Hyperbranching was observed in the *Fgrab1DN* strain. (**C**) *Fgrab1DN* displays a thicker cell wall compared to wild-type PH-1. CW: cell wall. Arrows mark the different layers: a. internal electron-dense layer, b. middle electron-dense layer, c. external electron-dense layer.

A previous study indicated that the deletion of Ypt1 (Rab1 homolog) leads to death in S. cerevisiae [22]. We therefore intended to delete FgRab1 for its functional analyses. Similar to the above observation in yeast, the *Fgrab1* mutant could not be obtained after several screenings, suggesting that the deletion of the protein is lethal to the fungus. As such, we used the point mutation strategy to modify the active site of the FgRab1 protein and successfully constructed *Fgrab1CA* (constitutively active, Q67L) (Figure S1A) and *Fgrab1DN* (dominant-negative, S22N) (Figure S1B) strains to analyze the gene function. We found that the growth of the *Fgrab1DN* strain was reduced by approximately 77% compared to the wild-type PH-1 strain, while the mycelial growth of the *Fgrab1CA* strain was only slightly reduced (Figure 2A). Moreover, mycelial branching was observed to increase in *Fgrab1DN* (Figure 2B), suggesting perturbation of the fungal polarity. To gain further insight into the effects of this mutation, the hyphae of the wild-type PH-1, *Fgrab1CA* and *Fgrab1DN* strains were stained with the fluorescent dye FM4-64 and observed under a confocal microscope. As shown in Figure 2C, the results showed an increase in the *Fgrab1DN* hyphal cell wall thickness compared to that of the wild type. However, the cell wall thickness of the *Fgrab1CA* strain was not affected. Taken together, these results indicate that FgRab1 is important for mycelial morphology and the polarized growth of *F. graminearum*.

Consistent with the above results, we observed during protoplast preparation of the various strains that the enzymatic cell wall degradation of the *Fgrab1DN* strain was very difficult to achieve. Two hours after treatment with the cell wall-degrading enzymes, most of the *Fgrab1DN* samples were still in their hyphal forms, and the protoplasts produced were significantly reduced compared to those obtained from the other strains (Figure S2). This supports the notion that the perturbation of FgRab1 function affects cell wall integrity in *F. graminearum*.

### 2.3. FgRab1 Is Involved in Regulating Conidiogenesis but Not Sexual Reproduction in F. graminearum

**Figure 3:**
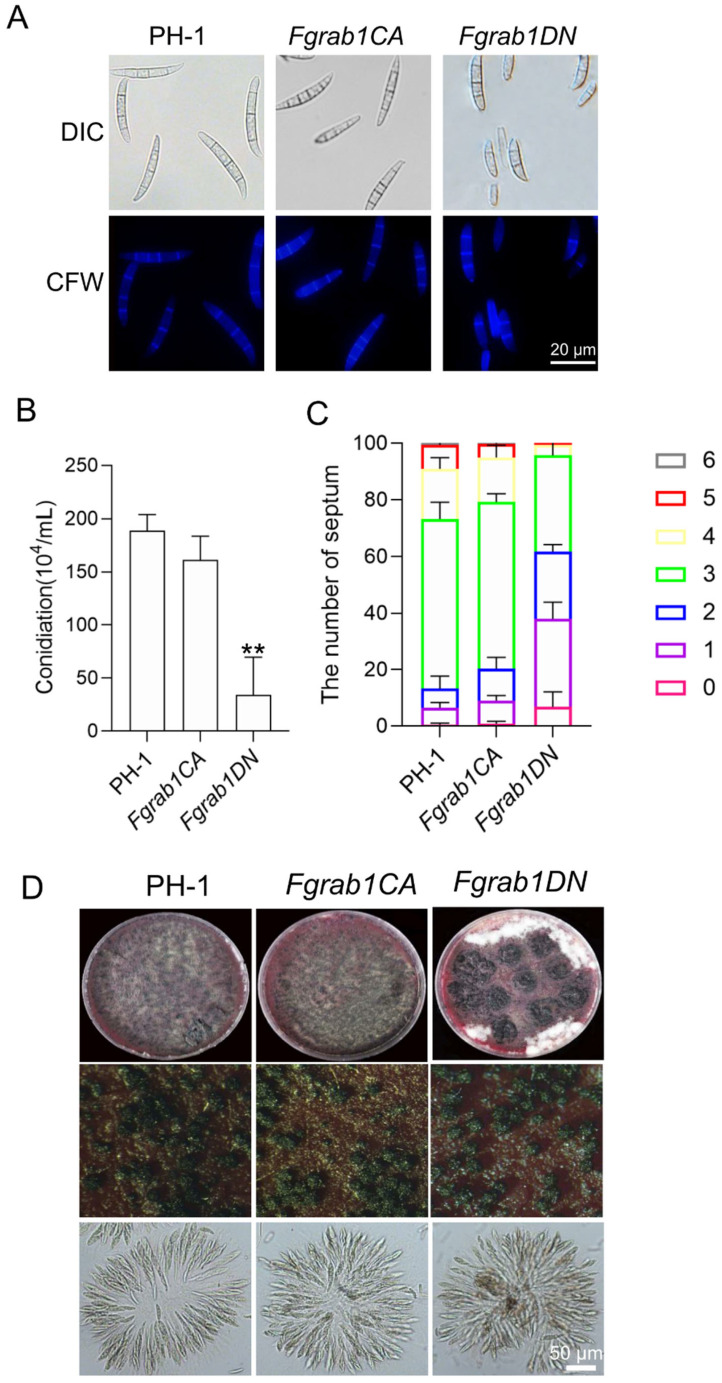
FgRab1 inactivation perturbs conidiation but not sexual reproduction in *F. graminearum*. (**A**) Conidial morphology of the wild-type PH-1, *Fgrab1CA* and *Fgrab1DN* strains, bar = 20 μm. (**B**) Conidia formation by the wild-type PH-1, *Fgrab1CA* and *Fgrab1DN* strains. Error bars represent SD from three replicates, and two-tailed Student’s *t*-test was used for paired comparison of the conidiation between PH-1 vs. *Fgrab1CA* and *Fgrab1DN* strains (**, *p* < 0.01). (**C**) The number of conidia septa of the indicated strains. Error bars represent SD from three replicates, which was used for paired comparison of the number of conidial septum between PH-1 vs. *Fgrab1CA* and *Fgrab1DN*. (**D**) Perithecia and ascospore formation by the indicated strains, bar = 50 μm.

To investigate the potential roles of FgRab1 in conidiogenesis, we cultivated wild-type PH-1, *Fgrab1CA* and *Fgrab1DN* in CMC media for 3 days to induce conidiation. We observed that the number of conidia produced by the *Fgrab1DN* strain was significantly decreased compared to PH-1 (Figure 3B). We further analyzed the distribution of the number of septa in each conidium and found that the number of septa was also significantly reduced. The number of septa in *Fgrab1DN* mainly ranged from zero to three, while that in the wild-type PH-1 conidia mainly concentrated around three (Figure 3C). These results suggest that FgRab1 is involved in conidia formation in *F. graminearum*.

To determine whether FgRab1 is involved in sexual reproduction, we inoculated the wild-type PH-1, *Fgrab1CA* and *Fgrab1DN* strains on carrot media to induce sexual reproduction. We found that both *Fgrab1CA* and *Fgrab1DN* strains could produce perithecia and ascospores as normally as the wild-type PH-1 strain (Figure 3D), suggesting that FgRab1 is dispensable for the sexual reproduction of *F. graminearum*.

### 2.4. FgRab1 Localizes to the Golgi, ER and Spitzenkörper

**Figure 4:**
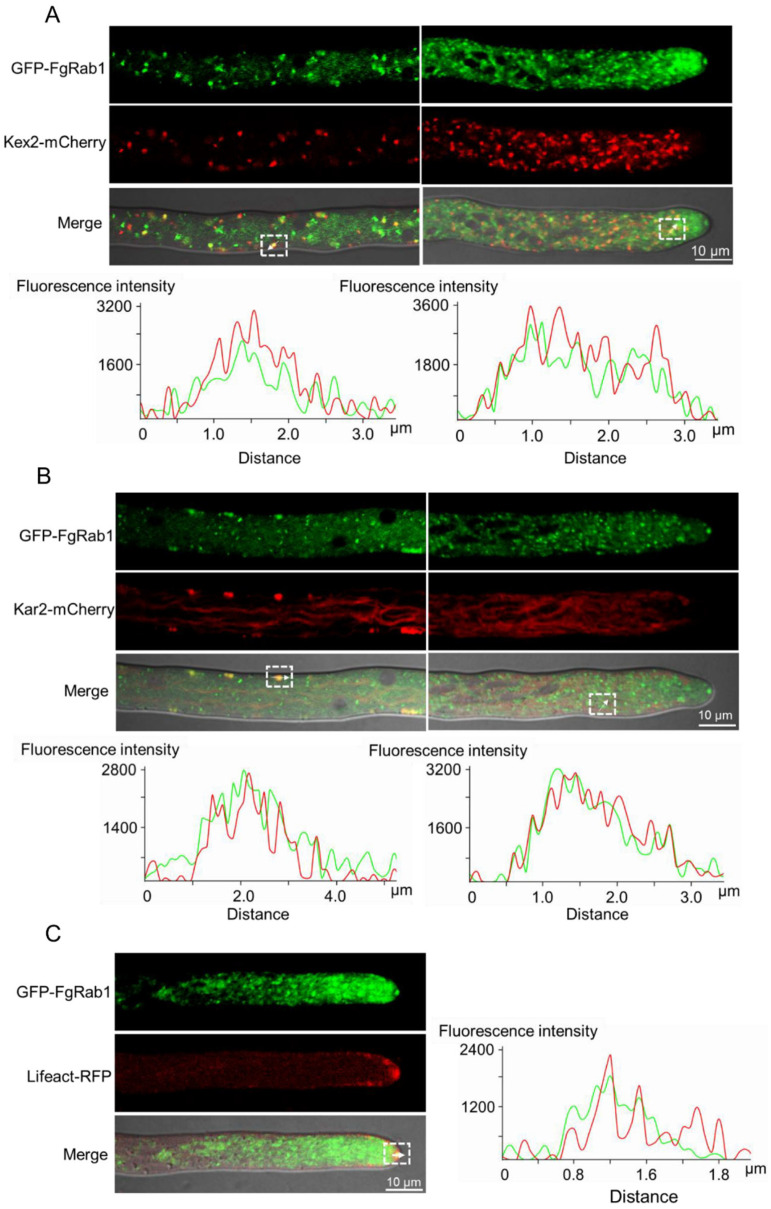
Subcellular localization of FgRab1. (**A**) GFP-FgRab1 co-localizes with the trans-Golgi marker Kex2-mCherry (marked by white arrows). A line scan graph was generated at the indicated position (arrow) to show the relative localization of GFP-FgRab1 (green) and Kex2-mCherry (red). (**B**) GFP-FgRab1 and Kar2-mCherry co-localize to the endoplasmic reticulum (ER) (marked by white arrows). A line scan graph was generated at the indicated position (arrow) to show the relative localization of GFP-FgRab1 (green) and Kar2-mCherry (red). (**C**) GFP-FgRab1 localizes to the Spitzenkörper (Spk) (marked by white arrows). A line scan graph was generated at the indicated position (arrow) to show the relative localization of GFP-FgRab1 (green) and Lifeact-RFP (red).

To investigate the subcellular localization of FgRab1, we transformed GFP-FgRab1 with Lifeact-RFP, FgKar2-mCherry and FgKex2-mCherry into the protoplasts of PH-1. As shown in Figure 4, GFP-FgRab1 partially co-localized with FgKar2-mCherry, FgKex2-mCherry and Lifeact-RFP in the hypha of *F. graminearum*. This suggests that FgRab1 is distributed around the Golgi, ER and Spitzenkörper in *F. graminearum*.

### 2.5. FgRab1 Is Required for Vesicle Trafficking and Fusion Process in F. graminearum

**Figure 5:**
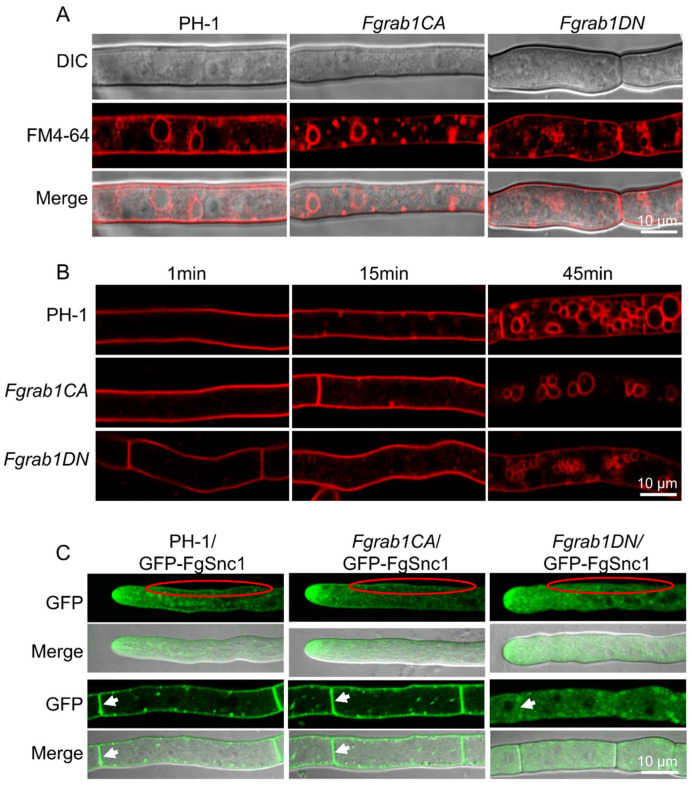
FgRab1 inactivation disrupts the transport of FgSnc1 to the plasma membrane and the fusion of vesicles with target membranes in *F. graminearum*. (**A**,**B**) FgRab1 is necessary for membrane fusion. (**C**) FgRab1 is required for the transport of FgSnc1 to the plasma membrane and septum. Red circles show the plasma membrane localization of GFP-FgSnc1, and white arrows show the septum localization of GFP-FgSnc1, bar = 10 μm.

In *S. cerevisiae*, Rab1/Ypt1 is mainly located in the Golgi and Spitzenkörper, and it plays a role in several transport pathways. To study the role of the FgRab1 protein in the endocytosis of *F. graminearum*, the mycelia of the wild type and mutants were stained with FM4-64 dye [41]. Within 1 min, obvious fluorescence could be observed on the plasma membranes of all the strains. After 15 min, a few red dots (FM4-64-labeled vesicles) appeared inside the three strains, indicating normal endocytosis, but at this time, a large amount of FM4-64 remained in the membrane on the surface of the mycelia. At 45 min post-staining, it was found that a large part of the FM4-64 had been internalized into the cell, and FM4-64 was particularly detected on the vacuolar membrane in PH-1 and *Fgrab1CA* strains, but only some tiny, fragmented vesicles were observed in the *Fgrab1DN* strain (Figure 5A, B).

To understand the influence of FgRab1 on vesicle transport processes, GFP-FgSnc1 was constructed and transformed into the protoplasts of PH-1, *Fgrab1CA* and *Fgrab1DN* strains. The localization of GFP-FgSnc1 was observed by confocal microscopy. As shown in Figure 5C, GFP-FgSnc1 localizes to the plasma membrane and concentrates at the Spitzenkörper in the growing hyphal cells of PH-1 and *Fgrab1CA*. However, in the *Fgrab1DN* strain, the plasma membrane and septum localization of this protein cannot be observed; instead, the GFP fluorescence is dispersed in the cytoplasm, though its localization to the hyphal tip was still present. These results suggest that the inactivation of FgRab1 affects the secretion of FgSnc1 from the Golgi to the plasma membrane and septum.

### 2.6. FgRab1 Is Required for Pathogenicity

**Figure 6:**
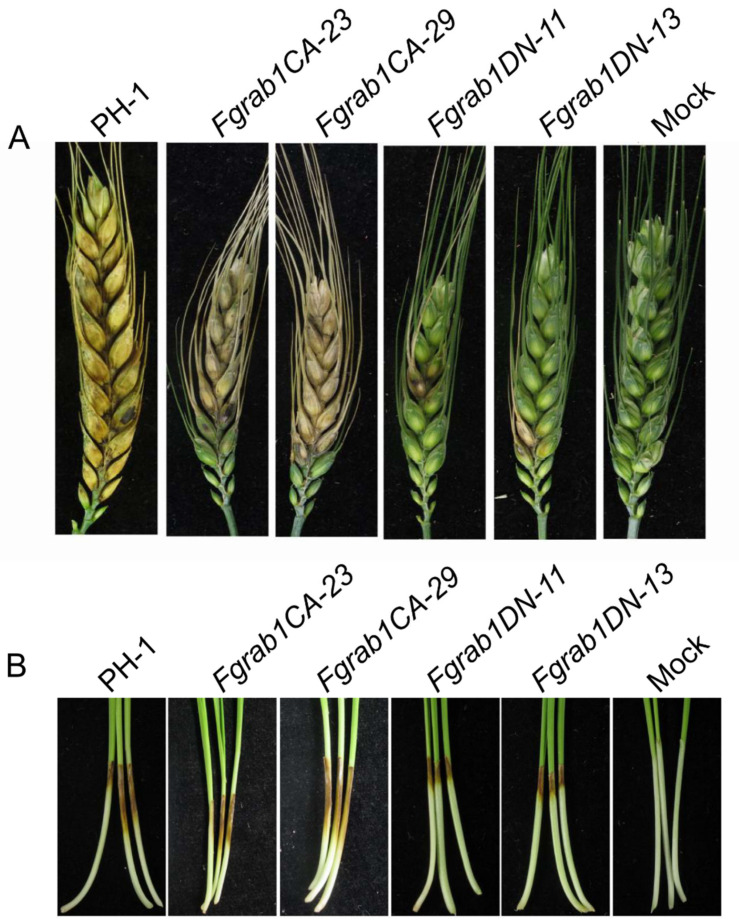
FgRab1 critically contributes to the pathogenicity of *F. graminearum*. (**A**) Pathogenicity of PH-1, *Fgrab1CA* and *Fgrab1DN* strains on flowering wheat heads. (**B**) Pathogenicity of PH-1, *Fgrab1CA*, *Fgrab1DN* strains on wheat coleoptiles.

To investigate the effects of *Fgrab1CA* and *Fgrab1DN* on the pathogenicity of the fungus, the mutants and the PH-1 control were inoculated on flowering wheat heads under moist conditions for 14 days. As shown in Figure 6A, the FBH symptoms due to *Fgrab1DN* infection on wheat heads were significantly lower than those observed due to PH-1 and *Fgrab1CA* infections. These results indicate that FgRab1 is required for normal pathogenicity of *F. graminearum*. Furthermore, infection assays on wheat coleoptiles yielded similar results (Figure 6B). Taken together, our data demonstrate that FgRab1 is important for *F. graminearum* virulence.

### 2.7. Inactivation of FgRab1 Prevents Autophagy in F. graminearum

**Figure 7:**
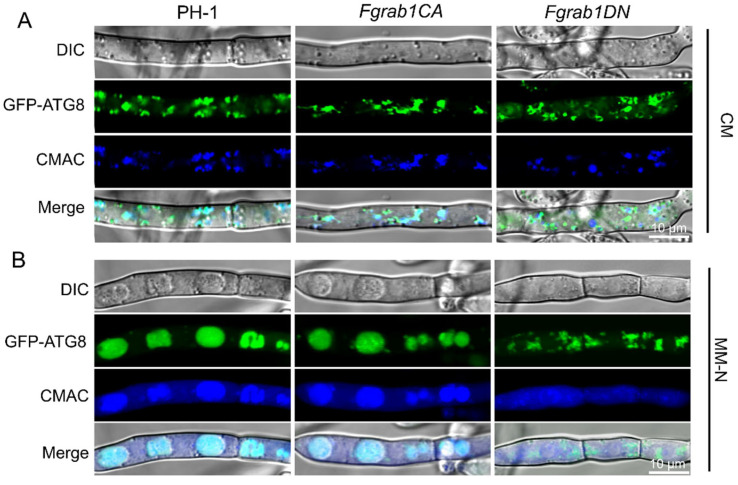
FgRab1 is required for autophagosome formation and autophagy in *F. graminearum*. Localization of GFP-FgAtg8 in PH-1, *Fgrab1CA* and *Fgrab1DN* strains in CM (**A**) or MM-N media (**B**). PH-1, *Fgrab1CA* and *Fgrab1DN* strains expressing GFP-FgAtg8 were grown in liquid CM medium at 28 °C for 16 h and then shifted to liquid MM-N medium containing 2 mM PMSF for 8 h to induce autophagy. Mycelia were stained with CMAC and examined under a confocal microscope. Bar = 10 μm.

Autophagy is a highly important and conserved intracellular process for the degradation and recycling of proteins and damaged organelles in eukaryotic organisms. GFP-FgAtg8 is widely used as a marker for reflecting the level of autophagy in numerous studies [39,46,47,48]. To investigate whether FgRab1 is involved in autophagy in *F. graminearum*, we transformed GFP-FgAtg8 into the protoplasts of the wild-type PH-1, *Fgrab1CA* and *Fgrab1DN* strains. We stained the transformed strains with the vacuole marker CMAC (7-amino-4-chloromethylcoumarin) and subjected the strains to confocal microscopy. We found that GFP-FgAtg8 localized to the cytosol in all the strains, forming punctate structures within the cytosol under nutrient-rich conditions (CM medium) (Figure 7A). Upon the induction of autophagy (growth of the strains on nutrient-deficient MM media for 8 h in the presence of 2 mM PMSF), more spots of GFP-FgAtg8 signals were observed in the vacuoles of PH-1 and *Fgrab1CA* mycelia, while the signals remained diffused within the cytosol in the *Fgrab1DN* strain (Figure 7B). This simply indicates that autophagy was prevented in the *Fgrab1DN* strain. These results indicate that FgRab1 is critically involved in autophagy in *F. graminearum.*

## 3. Discussion

Rabs, a subfamily of the Ras superfamily of small GTPases, are highly conserved in eukaryotic cells, which function as “molecular switches” between GTP- and GDP-bound states through interaction with two regulatory proteins, guanine nucleotide exchange factor (GEF) and GTPase-activating protein (GAP) [2,49]. Rab1 is a member of the Rab GTPase family, which is known to regulate cargo transport from the ER to the Golgi and intra-Golgi through different effectors, and it plays an essential role in the secretory pathway [50,51,52]. The intact *YPT1* gene has been shown to be essential for spore germination and the vegetative growth of yeast cells [22]. However, the biological functions of Rab1 in the phytopathogen are still largely unknown. In mice, Rab1 has GTP-binding sites of Ser22 (S) (preferential binding) and asparagine (N) (121) (low affinity) and glutamine (Q) with GDP-binding site 67 [53]. In this study, we found that the corresponding GTP-binding site of FgRab1 contains serine 22 (S) and asparagine 121 (N), while the GDP-binding site contains glutamine 67 (Q). Here, we used point mutation strategy to modify the active site of the FgRab1 protein, and we showed that FgRab1 is required for vegetative growth and pathogenicity in *F. graminearum*. Furthermore, we found that FgRab1 plays a crucial role in the secretory process from the Golgi to the plasma membrane and that it determines the effectiveness of vesicle fusion, as well as cell wall integrity. Furthermore, disrupting the functions of FgRab1 causes severe phenotypic defects, including fungal pathogenicity.

Rab1 is a key regulator of ER-to-Golgi transport, where the active Rab proteins are localized to the membranes of the endoplasmic reticulum (ER) and the Golgi apparatus [50]. In our study, we found that GFP-FgRab1 was mainly localized to the Golgi (Figure 4A), some to the ER (Figure 4B) and a small amount to the Spitzenkörper (Figure 4C). Snc1 is a v-SNARE protein that mediates the fusion of trans-Golgi network (TGN)-derived exocytic vesicles with the plasma membrane in budding yeast; the protein is also endocytosed from the cell surface and trafficked back to the TGN, where it can be repackaged into nascent secretory vesicles to facilitate another round of transport [54]. In our previous study, we demonstrated that FgGyp1, a GAP of FgRab1, is required for the FgSnc1-mediated fusion of secretory vesicles from the Golgi with the plasma membrane [42]. In wild-type PH-1, GFP-FgSnc1 is localized to the cytoplasm, Spitzenkörper, plasma membrane and septum [41,55]. However, in the present study, the fluorescence signal of FgSnc1 could not be observed at both the plasma membrane and septum of the *Fgrab1DN* strain, suggesting that the inactivation of FgRab1 affected the trafficking of FgSnc1 from the Golgi to the plasma membrane and septum. Taken together, our results indicated that the FgGyp1-FgRab1 cascade regulates the FgSnc1-mediated fusion of secretory vesicles from the Golgi with the plasma membrane.

In *Drosophila*, *omelette* (*omt*) is the orthologue of human Rab1. Golgi stacks appear “collapsed” in omt mutant cells, with abnormal Golgi complexes comprising fragmented cisternae and a few small stacks [52]. Moreover, in HeLa cells, an increase in Rab1b levels induces changes in Golgi size and gene expression, where the inhibition of Rab1 affects the stability of the Golgi apparatus structure, leading to the dispersal of the Golgi in the cytoplasm [56,57]. Therefore, we speculated that the inactivation of Rab1 in *F. graminearum* may affect the stability of the Golgi, which, in turn, could have been the reason why we observed diffused distribution of FgSnc1 in the cytoplasm, which affects the transport of FgSnc1 to the plasma membrane. It was reported in another study that inhibiting the function of Rab1 through the expression of the dominant-negative construct Rab1b N121I by siRNA transfection or overexpression of its GAP caused fragmentation of the Golgi [56]. Rab1 mediates the docking of ER-derived vesicles with the cis-Golgi compartment, where the knockdown of Rab1b alters vacuole growth, at least in part, by changing its fusogenic capacity with endocytic compartments [58]. To verify whether FgRab1 affects vesicle fusion in *F. graminearum*, we stained the mycelia of the wild-type PH-1, *Fgrab1CA* and *Fgrab1DN* strains with FM4-64, and we found that almost 90% of the mycelia from the *Fgrab1DN* strain could not form large vacuoles, and the vacuole pieces observed in the cytoplasm showed fragmentation (Figure 5C). This result suggests that the inactivation of FgRab1 affects the vesicle fusion process, leading to vacuolar fragmentation.

Polarized growth is fundamental for optimal survival, which requires a perfect coordination among the major cellular processes, such as cytoskeletal organization, secretion and endocytosis, which have to be controlled by the cell integrity pathway and regulated by the cell cycle [59]. As shown in our results, FgRab1 exhibited an obvious defect in endocytosis from the cell surface as well as trafficking back to the TGN, which further results in defects in vacuolar transport. In addition, we found that inactivating FgRab1 led to abnormal cell wall thickness, which affected the corresponding growth process. We speculated that FgRab1 may play an important role in the formation of the cell wall.

Autophagy is a highly important and conserved intracellular process for the degradation and recycling of subcellular materials in eukaryotes, and it is critical for vesicle formation, transport, tethering and fusion; hence, it is a strong determinant of fungal survival, reproduction and infection [47,60,61]. Autophagy plays a pivotal role in supplying nutrients to non-assimilating structures necessary for growth, and it is important for plant colonization in *F. graminearum* [47]. As vesicle-trafficking proteins, many Rab GTPases regulate various steps of autophagy. Rab1, Rab5, Rab7, Rab8A, Rab8B, Rab9, Rab11, Rab23, Rab24, Rab25, Rab32 and Rab33B are all essential for autophagy [60]. Ypt1 and its GEF TRAPPIII have been implicated in autophagy [62]. It has been reported that Gyp1, Gyp5 and Gyp8 function as the GAP of Ypt1 [42,62,63]. In yeast, Gyp1 has not only been proposed to negatively regulate Ypt1 at the Golgi but also acts as Atg8’s interacting partner for selective autophagy [62]. Rab1/Ypt1 is essential for autophagosome formation, and Trs85, a subunit of TRAPP complex III (the GEF for Ypt1), functions as a key player in channeling Ypt1 GEF and Ypt1 to the phagophore assembly site (PAS), where the key proteins are involved in autophagosome formation [64]. In this study, live-cell imaging showed abnormal autophagosomes in the *Fgrab1DN* strain during nitrogen starvation conditions, suggesting that autophagy was disrupted in *Fgrab1DN*. These results showed that FgRab1 plays an important role in autophagy.

In summary, FgRab1 is found herein to be important for pathogenicity in *F. graminearum*, and inactivation of the protein perturbs the fungal vegetative growth, autophagy and vesicle fusion with target membranes.

## 4. Materials and Methods

### 4.1. Fungal Strains and Culture Conditions

*F. graminearum* PH-1 (Taxonomy ID: 5518) was used as the wild-type strain and as a background for generating all the gene deletion mutants (Table S1). Fungal strains were cultured at 28 °C on complete medium (CM), which contained 6 g/L yeast extract, 6 g/L casamino acid, 10 g/L sucrose and 20 g/L agar (for solid medium) for mycelial growth. Carboxymethyl cellulose liquid medium (CMC) (1 g/L NH_4_NO_3_, 1 g/L yeast extract, 1 g/L KH_2_PO_4_, 0.5 g/L MgSO_4_·7H_2_O, 15 g/L carboxymethyl cellulose) was used for conidiation assays. Liquid trichothecene biosynthesis induction (TBI) medium (0.01 g/L FeSO_4_·7H_2_O, 0.05 g/L MgSO_4_·7H_2_O, 0.5 g/L KCl, 1 g/L KH_2_PO_4_, 0.0871 g/L arginine, 30 g/L sucrose, 200 μL/L trace elements, pH = 6.5) was used for trichothecene production analysis [9]. Carrot agar medium (400 g/L fresh carrot, 20 g/L agar) was used for inducing perithecia and ascospore formation. A 1000× trace element solution (22 g/L ZnSO_4_·7H_2_O, 11 g/L H_3_BO_3_, 5 g/L MnCl_2_·4H_2_O, 5 g/L FeSO_4_·7H_2_O, 1.7 g/L CoCl_2_·6H_2_O, 1.6 g/L CuSO_4_·5H_2_O, 1.5 g/L Na_2_MoO_4_·5H_2_O, 50 g/L EDTA·4Na) and 1000× vitamin solution (0.1 g/L biotin, 0.1 g/L pyridoxin, 0.1 g/L thiamine, 0.1 g/L riboflavin, 0.1 g/L p-aminobenzoic acid, 0.1 g/L nicotinic acid) were used for nitrogen-deficient minimal medium (MM-N, 0.52 g/L KCl, 0.312g/L MgSO_4_·7H_2_O, 1.52 g/L KH_2_PO_4_, 1 mL/L 1000× trace element solution, 1 mL/L 1000× vitamin solution). RNA was extracted from fresh mycelia harvested from 100 mL liquid CM cultured at 28 °C with constant shaking at 110 rpm for 3 days. The fresh mycelia were ground in liquid nitrogen into powder, and the total RNA was extracted using Eastep^TM^ Total RNA extraction Kit (Promega, Beijing). Each experiment was repeated three times.

### 4.2. Construction of Constitutively Active and Dominant-Negative Mutants

To generate dominant-negative and constitutively active *FgRAB1* mutants, we first used the primer pairs FgRab1DN1F/FgRab1DN1R and FgRab1DN2F/FgRab1DN2R, FgRab1DN1F/FgRabCA1R and FgRab1CA2F/FgRab1DN2R (Table S2) to amplify FgRAB1 DN1, DN2, CA1 and CA2, respectively, by PCR from the cDNA of PH-1, and the products were further subjected to splicing for overlap extension (SOE)-PCR to obtain the mutated constructs as previously reported [9]. The latter products were then cloned into *Bam*H I and *Not* I plasmids, downstream of the RP27 promoter on the pTE11-vector and verified by sequencing.

### 4.3. Quantitative Real-Time PCR

For qRT-PCR, the *Fgrab1DN*, *Fgrab1CA* and PH-1 strains were inoculated in liquid CM medium and incubated at 28 °C for 16 h. Total RNA was isolated from mycelia using Eastep^TM^ Total RNA extraction Kit (Promega, Beijing, China), and first-strand cDNA was synthesized using M-MLV (Moloney murine leukemia virus) reverse transcriptase (HiScript III RT SuperMix for qPCR (+gDNA wiper), Vazyme, Nanjing, China). Relative transcription levels were quantified using QuantiTect SYBRgreen PCR Master Mix (Takara, Kusatsu City, Japan), using the primer pairs (Supplementary Table S2). The tubulin beta chain gene (FGSG_09530) was used as the endogenous reference gene, and the data of relative quantification were calculated using the 2^−ΔΔCT^ method [65]. All experiments and qRT-PCR assays were repeated three times.

### 4.4. Asexual and Sexual Reproduction Assays

Mycelial agar blocks (5 mm in diameter) were inoculated into CMC liquid medium to assay asexual reproduction [9]. The number of conidia was determined 3 days after incubation at 28 °C, using a hemacytometer (QIUJING, Shanghai, China) under an Olympus BX53F microscope (Olympus, Tokyo, Japan). To induce sexual reproduction, mycelial agar blocks were placed on carrot agar and incubated at 28 °C for 5–7 days, after which aerial hyphae were removed, and the plates were pressed down gently with 1 mL of 2.5% sterile Tween 60 (Macklin, Shanghai, China). All of the sexual reproduction-induced cultures were incubated at 28 °C under black light (F20T8/BLB, wavelength: 365 nm, Danqi, Shanghai, China). The perithecia formed were photographed and recorded. Each experiment was independently repeated three times.

### 4.5. Pathogenicity Assays

Nearly the same quantities of fresh mycelia were inoculated into the center of flowering wheat heads, after which, the wheat heads were sprayed with sterile distilled water and covered with transparent polythene bags. After 7 days, the bags were removed, and after another 7 days, the wheat heads were cut off and photographed [9]. For conidial samples, 2 µL of 100 × 10^4^ cells/mL conidial suspension was inoculated in each coleoptile, and the observed symptoms were recorded after 7 days of inoculation.

### 4.6. Autophagy Assay

Autophagy was assayed by determining the expression of FgAtg8. We transformed the GFP-FgAtg8 construct into the PH-1, *Fgrab1CA* and *Fgrab1DN* strains. The transformants were cultured in liquid CMC for 3 days to obtain their respective conidia. The conidia were harvested and transferred into fresh liquid CM cultures and further incubated for 16 h at 28 °C, with shaking at 180 rpm. The conidia were then transferred into “starvation media”, that is, nitrogen-deficient minimal media (MM-N) containing 2 mM phenylmethylsulfonyl fluoride (PMSF), and cultured for 8 h at 28 °C, 150 rpm. Mycelia were stained with 7-amino-4-chloromethylcoumarin (CMAC) for 30 min and observed under fluorescence confocal microscope.

### 4.7. Live-Cell Imaging of F. graminearum

Confocal microscopy was used for time-lapse or live-cell fluorescence imaging using a Nikon A1R laser scanning confocal microscope system (Nikon, Tokyo, Japan). Alternatively, an Olympus BX51 microscope (Olympus, Tokyo, Japan) was used for conventional epifluorescence and differential interference contrast (DIC). Elapsed time is indicated in seconds. We used 405 nm light (Em. 452/45 nm) for Calcofluor white and CMAC excitations. GFP excitation was performed using 488 nm light (Em. 525/40 nm), and FM4-64 and mCherry excitations were performed using 561 nm light (Em. 607/36 nm) [9].

### 4.8. Statistical Analysis

GraphPad Prism 8 (Scientific Software, San Diego, CA, USA) was used to calculate the standard error of the mean (sem) for all values shown. *p* values were calculated with a two-tailed Student’s *t*-test using Microsoft Excel (Microsoft, Redmond, WA, USA).

## Data Availability

Any data or material that support the findings of this study can be made available by the corresponding author upon request.

## Supplementary Materials

The following supporting information can be downloaded at: https://www.mdpi.com/article/10.3390/ijms23020895/s1.
