# Supplementary material for: The Small GTPase FgRab1 Plays Indispensable Roles in the Vegetative Growth, Vesicle Fusion, Autophagy and Pathogenicity of Fusarium graminearum
Source: Int J Mol Sci. 2022 Jan 14;23(2):895. doi: 10.3390/ijms23020895 (PMC8776137; doi:10.3390/ijms23020895)
Supplement: Supplementary file 1 [file ijms-23-00895-s001.zip › ijms-1540935-supplementary.pdf]

International Journal of Molecular Sciences

MDPI

Supplementary material

The Small GTPase FgRab1 Plays Indispensable Roles in the Vegetative Growth, Vesicle Fusion, Autophagy and Pathogenicity of *Fusarium graminearum*

Yanping Yuan [1], Meiru Zhang [1], Jingjing Li [1], Chengdong Yang [1], Yakubu Saddeeq Abubakar [1,2], Xin Chen [3], Wenhui Zheng [3], Zonghua Wang [1,4], Huawei Zheng [4,*] and Jie Zhou [1,*]

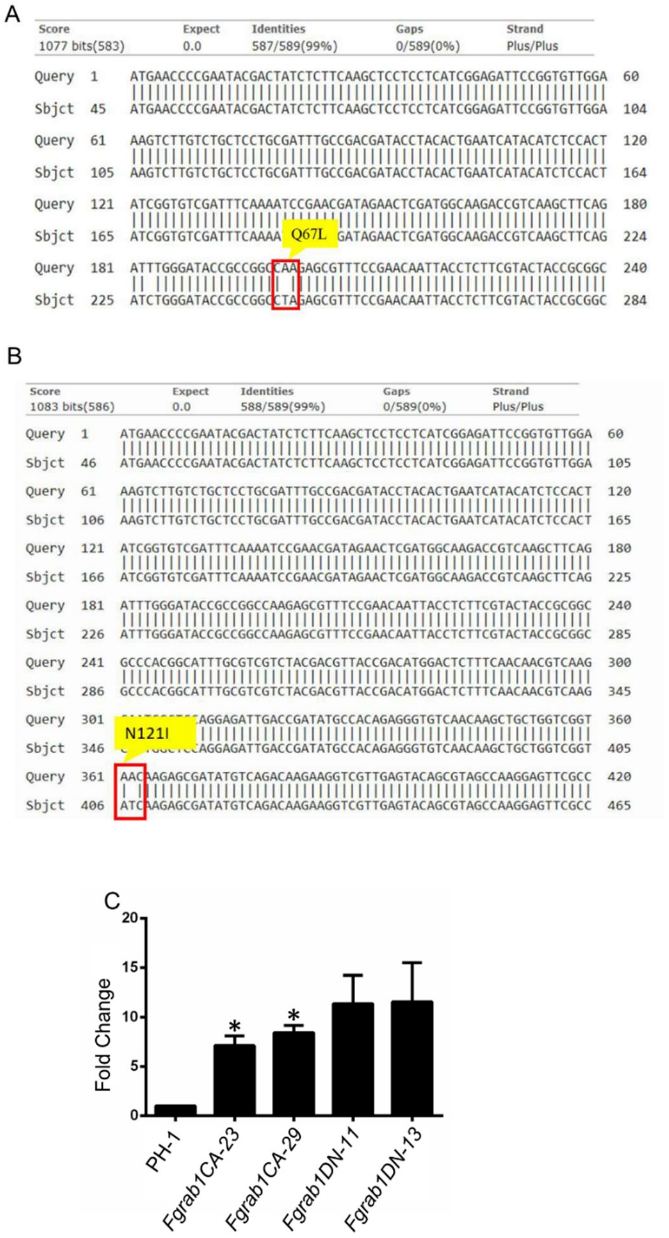

Figure S1. Construction and identification of *Fgrab1CA* and *Fgrab1DN* strains: (A, B) Sequencing results for positive transformants of *Fgrab1CA* and *Fgrab1DN* strains. (C) Relative expression levels of *FgRAB1* in *Fgrab1CA* and *Fgrab1DN* strains，*P＜0.05, **P＜0.01 according to *t*-test.

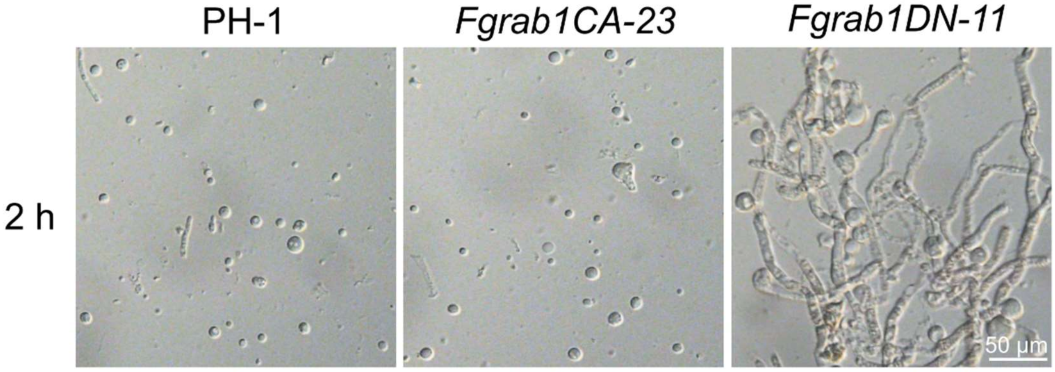

Figure S2. Light microscopic examination of the protoplasts released by the indicated strains after 2 h.

Table S1. Wild type (PH-1) and mutant strains of fungi used in this study.

Strain	Description	Reference
PH-1	Wild type	
Fgrab1CA-23	Constitutively active in PH-1	This study
Fgrab1DN-11	Dominant negative in PH-1	This study
FgGFP-Rab1/ER-mCherry	FgGFP-Rab1 and ER-mCherry are co- express in PH-1	This study
FgGFP-Rab1/TGN-mCherry	FgGFP-Rab1 and TGN-mCherry are co- express in PH-1	This study
FgGFP-Rab1/Lifeactin-RFP	FgGFP-Rab1 and Lifeactin-RFP are co- express in PH-1	This study
PH-1: ER-mCherry	PH-1 strain expressing the ER-mCherry construct	This study
Fgrab1CA: ER-mCherry	*Fgrab1CA* strain expressing the ER-mCherry construct	This study
Fgrab1DN: ER-mCherry	*Fgrab1DN* strain expressing the ER-mCherry construct	This study
PH-1: GFP-FgSnc1	PH-1 strain expressing the FgSnc1-GFP construct	This study
Fgrab1CA: GFP-FgSnc1	*Fgrab1CA* strain expressing the FgSnc1-GFP construct	This study
Fgrab1DN: GFP-FgSnc1	*Fgrab1DN* strain expressing the FgSnc1GFP construct	This study

Table S2. Primers used in this study.

Primers	（5′ → 3′）Sequences	Function
FGRab1DN1F	CGGGATCCATGAACCCCGAATACGACTA	For *Fgrab1DN* construction
FGRab1DN1R	TGTCTGACATATCGCTCTTGATACCGACCAGCAGCTTG	
FGRab1DN2F	CAAGCTGCTGGTCGGTATCAAGAGCGATATGTCAGACA	
FGRab1DN2R	ATAAGAATGCGGCCGCTTAGCAGCAGCTGTTGTTGG	
FGRab1CA1F	AAAATGGCTCGAGGGGGATCCATGAACCCCGAATACGACTA	For *Fgrab1CA* construction
FGRab1CA1R	TTCGGAAACGCTCTAGGCCGGCGGTATCCCAAATCTGAAG	

FGRab1CA2F	TGGGATACCGCCGGCCTAGAGCGTTTCCG AACAATTACCT	
FGRab1CA2R	TGAGTGGAATGATGCGGCCGCTTAGCAG CAGCTGTTGTTGG	
pTE11-F2	AGCCGACTATACTGAGCACCAACGC	For *Fgrab1DN*
pTE11-R2	TAAAACACTGTAACCCGCAACGAAG	construction
Fgrab1 QF	AACCCCGAATACGACTA	qRT-PCR primer
Fgrab1 QR	TTCAGTGTAGGTATCGTC	for *FgRAB1*
β-Tubulin F	TCTGACTTCAGGAATGGTCGTTAC	Inference primers
β-Tubulin R	AGCGGTCTGGATGTTGTTGG	for qRT-PCR
